# β-blockers and breast cancer survival by molecular subtypes: a population-based cohort study and meta-analysis

**DOI:** 10.1038/s41416-022-01891-7

**Published:** 2022-06-20

**Authors:** L. Lukas Løfling, Nathalie C. Støer, Erica K. Sloan, Aeson Chang, Sara Gandini, Giske Ursin, Edoardo Botteri

**Affiliations:** 1 Department of Research, Cancer Registry of Norway, Oslo, Norway; 2 Norwegian Research Centre for Women’s Health, Women’s Clinic, Oslo University Hospital, Oslo, Norway; 3 Drug Discovery Biology Theme, Monash Institute of Pharmaceutical Science, Monash University, Parkville, VIC Australia; 4 Department of Experimental Oncology, European Institute of Oncology IRCCS, Milan, Italy; 5 Cancer Registry of Norway, Oslo, Norway; 6 Department of Nutrition, Institute of Basic Medical Sciences, University of Oslo, Oslo, Norway; 7 Department of Preventive Medicine, University of Southern California, Los Angeles, CA USA; 8 Section for Colorectal Cancer Screening, Cancer Registry of Norway, Oslo, Norway

**Keywords:** Cancer epidemiology, Breast cancer

## Abstract

**Background:**

The association between use of β-blockers and breast cancer (BC) prognosis has been investigated in several observational studies, with conflicting results. We performed a nationwide cohort study and a meta-analysis to investigate the association, and assess if it varied between molecular subtypes of BC.

**Methods:**

We identified women aged ≥50 years with BC diagnosed between 2004 and 2018 in Norway. We used Cox regression models to estimate the association between β-blocker use at diagnosis and BC-specific survival, overall and by molecular subtype. We performed a meta-analysis of observational studies that reported molecular subtype-specific estimates of this association.

**Results:**

We included 30,060 women, of which 4461 (15%) used β-blockers. After a median follow-up of 5.1 years, 2826 (9%) died of BC. Overall, β-blocker use was not associated with BC-specific survival (hazard ratio [HR] = 1.07; 95% confidence interval [CI]: 0.97–1.19). We found an association only in triple-negative BC (TNBC) patients (HR = 0.66; 95% CI: 0.47–0.91). This was confirmed in the meta-analysis: β-blocker use was associated with progression/recurrence-free (HR = 0.58; 95% CI: 0.38–0.89) and BC-specific survival (HR = 0.74; 95% CI: 0.55–1.00) in TNBC patients only.

**Conclusion:**

In our cohort of BC patients and in the meta-analysis, β-blocker use was associated with prolonged BC-specific survival only in TNBC patients.

## Background

Breast cancer (BC) is the most common cancer and a leading cause of cancer death among women worldwide [1]. BC survival has improved in the last two decades due to more accurate treatment strategies, and the introduction of new and effective systemic and targeted therapies [2, 3]. However, the prognosis of some BCs (e.g. advanced BC and triple-negative BC [TNBC]) remains poor, because of their unfavourable biology and lack of targeted therapies [4].

β-adrenergic signalling is involved in various processes during tumour progression, including suppression of antitumor immunity [5, 6], vasculature remodelling [7, 8], and tumour cell dissemination [9, 10]. Inhibition of these processes by blocking β-adrenergic receptors using β-blocker drugs reduces cancer progression in preclinical models of cancer [5, 7, 9, 11, 12]. β-blocker drugs have been used since the 1950s to treat cardiovascular conditions. Recent observational studies have investigated β-blockers as a medication that potentially can be repurposed in the treatment of cancer, especially BC [13]. However, the published results are inconsistent, plausibly due to the heterogeneity of the different study populations in terms of tumour characteristics, in particular molecular subtype [14–20].

Little is known about the effect of β-blockers on BC prognosis according to tumour characteristics. To address this, we investigated the association between use of β-blockers and BC-specific survival in a cohort of BC patients, analysed as a whole and also stratified by stage, molecular subtype, receptor status and Ki-67 status. To validate the findings of the cohort study and provide a summary estimate of the association between use of β-blockers and BC prognosis based on the available literature, we conducted a systematic review and meta-analysis of those observational studies (including the original cohort study described here) that reported molecular subtype-specific estimates of the association.

## Methods

### Study design and study population

We performed a nationwide population-based cohort study of women with BC to assess the association between use of β-blockers at the time of diagnosis and BC-specific survival. Data on patients registered with a cancer diagnosis in the Cancer Registry of Norway between 2004 and 2018 were linked with data from the Norwegian Prescription Database, the Cause of Death Registry, the Population Registry, and different socioeconomic registries at Statistics Norway using the unique personal identification number given to all residents in Norway [21]. For this study, we identified women residing in Norway who received a diagnosis of primary invasive BC (International Classification of Diseases 10th revision [ICD-10] code: C50) between April 2004 and December 2018. The Norwegian Prescription Database started recording data on filled prescriptions in January 2004, and April 2004 was picked as the starting date to ensure that all individuals were covered by the Norwegian Prescription Database for 3 months prior to their BC diagnosis.

The inclusion was limited to patients aged ≥50 years at diagnosis to obtain more comparable age distributions in users and non-users of β-blockers, and to get a more homogeneous study population by including mostly post-menopausal women. We further limited the inclusion to breast carcinomas (ICD for Oncology 3rd revision [ICD-O-3] morphology codes: 801–823, 825–867, or 894) with no known previous diagnosis of invasive cancer (except invasive non-melanoma skin cancer [ICD-10: C44]).

#### Cancer registry of Norway

The Cancer Registry of Norway has recorded incident cancer cases in Norway since 1953 [3]. The completeness of the registry for BC is estimated at >99%, and >99% of the cases have been morphologically verified [22].

Tumour grade was based on the sixth digit in the ICD-O-3 morphology code recorded in the Cancer Registry of Norway and categorised as I (low), II (intermediate), and III (high) [23]. Information on oestrogen receptor (ER) status, progesterone receptor (PR) status, and human epidermal growth factor receptor 2 (HER2) status assessed by immunohistochemistry or in-situ hybridisation is routinely retrieved from pathology reports and registered by the Cancer Registry of Norway [4]. Since 2012, the Cancer Registry of Norway has routinely collected information on Ki-67 status, reported as a percentage of Ki-67 positive tumour cells by immunohistochemistry. Using the information on receptor status and Ki-67, we categorised BC into five molecular subtypes; luminal A (ER+ and/or PR+, HER2−, Ki-67 ≤ 14), luminal B HER2− (ER+ and/or PR+, HER2-, Ki-67 > 14), luminal B HER2+ (ER+ and/or PR+, HER2+), HER2+ (ER−, PR−, HER2+), and TNBC (ER−, PR−, HER2−). In the case of missing Ki-67, we used tumour grade I for luminal A, and II-III for luminal B HER2− [24].

Histology was based on the first three digits in the ICD-O-3 morphology code and categorised as ductal carcinoma (code 850), lobular carcinoma (code 852), and other forms of carcinoma. Disease stage, and T- and N-stages were retrieved by the Cancer Registry of Norway from pathology reports or clinical notifications. Clinical stage was used for patients who received neoadjuvant treatment, while pathological stage was used for those who did not. The disease stage was categorised as local, regional, or distant according to the United States National Cancer Institute’s Surveillance, Epidemiology, and End Results (SEER) Programme [25]. The T- and N-stages were classified and categorised according to the American Joint Committee on Cancer classification [26].

#### Other registries

Information on filled prescriptions was obtained from the Norwegian Prescription Database [27], and cause and date of death from the Cause of Death Registry [28]. Date of emigration was obtained from the Population Registry at Statistics Norway [29]. Data on education level, marital status, number of children and country of origin (based on country of origin going back three generations) was obtained from Statistics Norway.

### Exposure assessment

To assess use of β-blockers at diagnosis of BC, individuals were considered to be users of β-blockers if they had filled a prescription for a β-blocker (Anatomical Therapeutic Chemical [ATC] code C07) within 3 months before the diagnosis of BC. Use at diagnosis was chosen as the exposure of interest because of the hypothesis that β-blockers might exert its protective effect through an interaction with the BC treatment in the diagnostic period [30, 31]. Use of β-blockers was analysed as any type of β-blocker, and separately as selective β-blockers (ATC codes C07AB, C07BB) and non-selective β-blockers (ATC codes C07AA, C07AG). Individuals who filled both non-selective β-blockers and selective β-blockers were analysed as exposed to non-selective β-blockers only.

### Statistical analysis

#### Cohort study

To estimate hazard ratios (HR) for the association between use of β-blockers and BC-specific survival, and associated 95% confidence intervals (CI), we used Cox proportional hazard regression models with time since BC diagnosis as the underlying time scale. Follow-up time started at the date of the BC diagnosis and ended at the date of death due to BC, death due to other causes, emigration, or 31 December 2018, whichever came first. Schoenfeld residuals were used to test the proportional hazards assumption. We used a Cox model adjusted for possible confounders selected a priori based on existing evidence: age (continuous), molecular subtype, histology, year of BC diagnosis (continuous), education (primary, secondary, higher, missing), marital status (not married/not in partnership, married/in partnership, missing), number of children (0, 1, 2, ≥3), country of origin (Norway, other Nordic countries [i.e. Sweden, Denmark, Finland, and Iceland], and rest of the world), and use of (≥1 filled prescription) angiotensin-converting enzyme inhibitors (ACEI), angiotensin receptor blockers (ARB), calcium channel blockers (CCB), diuretics, low dose aspirin, cyclooxygenase2 (COX2) inhibitors, statins, and antidiabetics within 3 months before the BC diagnosis [4, 32–35]. The ATC codes used to identify use of the medications are listed in Supplementary Table 1. Missing information on covariates was handled as a separate category in the variable. Also, we estimated Kaplan–Meier curves of the BC-specific survival by use of β-blocker, stratified by age (≤median age and >median age of users).

Analyses were stratified by molecular subtypes, receptor status, Ki-67, and stage. Analyses that stratified by both molecular subtype and stage had low power and were only adjusted for the significant variables (*p* value <0.05) in any of the three models stratified by stage (i.e. age, year of BC diagnosis, education, marital status, number of children, histology, and use of diuretics). We assured that none of the excluded covariates changed the HR for use of β-blockers by >5%.

#### Sensitivity analyses

To better address confounding by indication, we used a reference group of patients who did not fill a prescription of β-blockers but filled a prescription of other antihypertensive medications (ACEI, ARB, CCB, diuretics). We also assessed if the associations were sensitive to the length of the exposure assessment window, and instead of having 3 months before diagnosis, we used exposure assessment windows of 4 and 6 months before diagnosis. In these sensitivity analyses, patients diagnosed from May 2004 (for the 4 months exposure assessment window) and July 2004 (for the 6 months exposure assessment window) onwards, were included, instead of April 2004.

To assess the influence of missing values, we performed a complete-case analysis including only women who had no missing value for any of the adjustment variables and a multiple imputation analysis. In the latter, we used a chained equation, assuming missing values were missing at random [36]. The multiple imputation model included the outcome, exposure and adjustment variables. We imputed 10 datasets and used Rubin’s rules to combine the estimates and standard errors.

All tests were two sided with a 5% significance level. All statistical analyses were performed using R version 3.4.4 (http://cran.r-project.org), and the R package *mice* was used for the multiple imputations [36].

#### Systematic review and meta-analysis

We performed a systematic review and meta-analysis of observational studies (including conference abstracts) that reported molecular subtype-specific estimates of the association between use of β-blockers and BC prognosis. We searched PubMed and Web of Science (Supplementary Table 2), without any restriction on language or publication date. Two of the authors (LLL and EB) independently searched and selected the articles and extracted the data. We screened titles and abstracts, and the full-text was retrieved if the article potentially reported original estimates of the association of interest. An article was included if it reported molecular subtype-specific estimates with a measure of uncertainty (i.e. CI, *p* value or standard error). Where multiple articles described overlapping study populations, we included the article reporting the largest study population. In addition, we reviewed articles that were included in previously published reviews and meta-analyses, reference lists of the included studies, and we performed a non-systematic search in Google Scholar.

The study by Santala et al. reported estimates for both pre-diagnostic use and post-diagnostic use of β-blockers [20], we chose the estimates for pre-diagnostic use. In the case of multiple estimates of the same association, we chose the estimate deriving from the most adjusted model. Therefore, for the original cohort study reported here and the study by Lorona et al. [19] we used the estimates that compared users of β-blockers to users of other antihypertensive medications, because those estimates took into account the potential effect of hypertension. The estimates from the other studies used all non-users of β-blockers as comparison group.

In a sensitivity analysis, we changed the estimates for the original cohort study reported here and the study by Lorona et al. [19] to estimates that compared users of β-blockers to all non-users of β-blockers, while the estimates from the other studies remained the same.

Pooled HRs and 95% CIs were calculated using the random effects model [37]. One analysis combined estimates of progression-free survival (PFS) and recurrence-free survival (RFS), while a second analysis included estimates of BC-specific survival. We measured heterogeneity using *I*^2^ [38]. Publication bias was evaluated graphically with a funnel plot and assessed with Egger’s test for the molecular subtypes with significant associations in the meta-analysis [39]. The quality of the studies included in the systematic review was assessed using the Newcastle–Ottawa scale [40].

## Results

We identified 41,764 women diagnosed with primary invasive BC (Supplementary Fig. 1). Of these, 11,704 patients were excluded because they had cancers that were not carcinoma (*n* = 254), were <50 years at diagnosis (*n* = 9006) or had previous cancer (*n* = 2444). In total, 30,060 patients were included in the study population. Of these, 4461 (15%) used β-blockers at the time of the BC diagnosis (577 [2%] non-selective and 3884 [13%] selective β-blockers), and 25,599 (85%) did not. The median age at diagnosis was 67 years for users of non-selective β-blockers, 69 years for users of selective β-blockers, and 62 years for non-users of β-blockers (Table 1). Users of β-blockers were more likely to be users of other medications at the time of the BC diagnosis, including other antihypertensive medications. After a median follow-up of 5.1 years (1st quartile: 2.3, 3rd quartile: 9.0), 2826 (9%) patients died from BC and 2575 (9%) died from other causes.

**Table 1:** Baseline characteristics of patients diagnosed with primary invasive breast cancer (*n* = 30,060), from 2004 to 2018 in Norway.

	No BB use (*N* = 25,599)	Non-selective BB (*N* = 577)	Selective BB (*N* = 3884)
Age (years)			
Median [q1, q3]	62 [56, 69]	67 [61, 75]	69 [63, 78]
Attained education			
Primary	6188 (24.2)	179 (31.0)	1416 (36.5)
Secondary	12,220 (47.7)	296 (51.3)	1863 (48.0)
Higher	7009 (27.4)	97 (16.8)	587 (15.1)
Missing	182 (0.7)	5 (0.9)	18 (0.5)
Marital status			
Not married/not in partnership	10,818 (42.3)	278 (48.2)	1841 (47.4)
Married/in partnership	14,720 (57.5)	299 (51.8)	2036 (52.4)
Missing	61 (0.2)	0 (0.0)	7 (0.2)
Children			
0	3020 (11.8)	67 (11.6)	404 (10.4)
1	3694 (14.4)	77 (13.3)	514 (13.2)
2	10,449 (40.8)	226 (39.2)	1472 (37.9)
≥3	8436 (33.0)	207 (35.9)	1494 (38.5)
Country of origin			
Norway	23,282 (90.9)	536 (92.9)	3618 (93.2)
Another Nordic country^a^	742 (2.9)	17 (2.9)	91 (2.3)
Rest of the world	1575 (6.2)	24 (4.2)	175 (4.5)
Stage			
Localised	15,578 (60.9)	342 (59.3)	2145 (55.2)
Regional	7609 (29.7)	167 (28.9)	1206 (31.1)
Distant	908 (3.5)	29 (5.0)	185 (4.8)
Missing	1504 (5.9)	39 (6.8)	348 (9.0)
T-descriptor (dimension)			
1	15,249 (59.6)	300 (52.0)	1954 (50.3)
2	6243 (24.4)	170 (29.5)	1049 (27.0)
3	797 (3.1)	20 (3.5)	138 (3.6)
4	502 (2.0)	11 (1.9)	104 (2.7)
Missing	2808 (11.0)	76 (13.2)	639 (16.5)
N-descriptor (lymph nodal involvement)			
0	15,961 (62.4)	352 (61.0)	2178 (56.1)
1	6291 (24.6)	132 (22.9)	945 (24.3)
2	746 (2.9)	25 (4.3)	129 (3.3)
3	424 (1.7)	11 (1.9)	71 (1.8)
Missing	2177 (8.5)	57 (9.9)	561 (14.4)
Histology			
Ductal Carcinoma	20,170 (78.8)	456 (79.0)	3007 (77.4)
Lobular Carcinoma	3284 (12.8)	87 (15.1)	468 (12.0)
Other carcinoma	2145 (8.4)	34 (5.9)	409 (10.5)
Tumour grade			
I	5737 (22.4)	122 (21.1)	784 (20.2)
II	11,736 (45.8)	276 (47.8)	1747 (45.0)
III	5962 (23.3)	125 (21.7)	890 (22.9)
Missing	2164 (8.5)	54 (9.4)	463 (11.9)
Ki-67			
Median [q1, q3]	21 [12, 37]	23 [12, 36]	24 [14, 39]
Missing	12,522 (48.9)	346 (60.0)	2020 (52.0)
Molecular subtype			
Luminal A	5612 (21.9)	119 (20.6)	736 (18.9)
Luminal B HER2 negative	10,780 (42.1)	252 (43.7)	1637 (42.1)
Luminal B HER2 positive	2000 (7.8)	45 (7.8)	272 (7.0)
HER2 positive	940 (3.7)	12 (2.1)	134 (3.5)
Triple-negative	1775 (6.9)	31 (5.4)	281 (7.2)
Missing	4492 (17.5)	118 (20.5)	824 (21.2)
Concomitant medication use			
Angiotensin-converting enzyme inhibitors^b^	981 (3.8)	65 (11.3)	627 (16.1)
Angiotensin receptor blockers^b^	3779 (14.8)	191 (33.1)	1389 (35.8)
Calcium channel blockers^b^	1959 (7.7)	121 (21.0)	970 (25.0)
Diuretics^b^	3406 (13.3)	226 (39.2)	1741 (44.8)
Low dose aspirin	2254 (8.8)	147 (25.5)	1368 (35.2)
COX2 inhibitors	876 (3.4)	11 (1.9)	75 (1.9)
Statins	3720 (14.5)	194 (33.6)	1711 (44.1)
Antidiabetics	1028 (4.0)	71 (12.3)	458 (11.8)
Follow-up time (years)			
Median [q1, q3]	5.2 [2.3, 9.1]	5.7 [2.9, 9.6]	4.4 [1.9, 8]

*BB* β-blocker, *q1* 1st quartile, *q3* 3rd quartile, *HER2* human epidermal growth factor receptor 2, *COX2* cyclooxygenase2.

^a^Includes Sweden, Denmark, Finland, and Iceland.

^b^Antihypertensives other than β-blockers.

### β-blockers and BC mortality

**Fig. 1:**
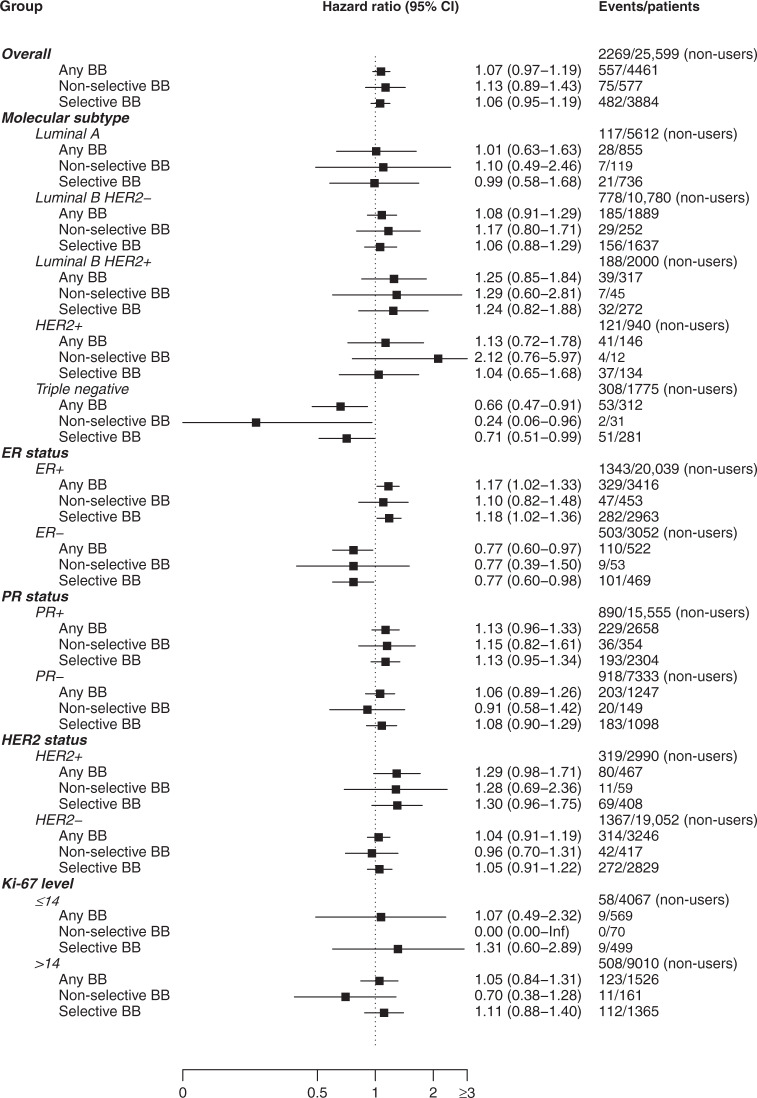
Hazard ratios (HR) and 95% confidence intervals (CI) for the association between use of β-blockers (filled prescription within 3 months before the diagnosis) and breast cancer-specific survival, overall and stratified by molecular subtype, receptor status, Ki-67, from 2004 to 2018 in Norway. Comparison group is all non-users of β-blockers. CI confidence interval, BB β-blocker, ER oestrogen receptor, PR progesterone receptor, HER2 human epidermal growth factor receptor 2.

**Fig. 2:**
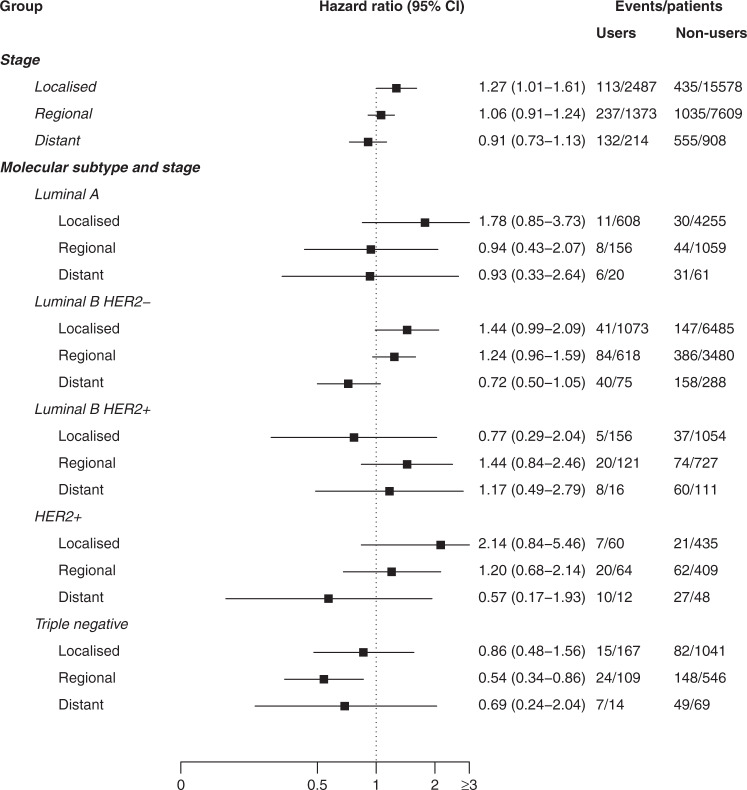
Hazard ratios (HR) and 95% confidence intervals (CI) for the association between use of β-blockers (filled prescription within 3 months before the diagnosis) and breast cancer-specific survival, stratified by stage and molecular subtype further stratified by stage, from 2004 to 2018 in Norway. Comparison group is all non-users of β-blockers. CI confidence interval, BB β-blocker, HER2 human epidermal growth factor receptor 2.

BC was the underlying cause of death for 557 (12%) users of β-blockers and for 2269 (9%) non-users. In multivariable analysis, the HR for the association between use of any type of β-blockers and BC-specific survival was 1.07 (95% CI: 0.97–1.19) (Fig. 1). The HR for non-selective β-blockers was 1.13 (95% CI: 0.89–1.43), and for selective β-blockers was 1.06 (95% CI: 0.95–1.19). When stratified by molecular subtype, use of β-blockers was associated with BC-specific survival only in patients with TNBC: the HRs were 0.66 (95% CI: 0.47–0.91) for use of any type of β-blocker, 0.24 (95% CI: 0.06–0.96) for use of non-selective β-blocker, and 0.71 (95% CI: 0.51–0.99) for use of selective β-blockers. When stratified by ER status, HRs for use of any type of β-blocker were estimated at 1.17 (95% CI: 1.02–1.33) among patients with ER+, and 0.77 (95% CI: 0.60–0.97) among patients with ER- BC. We found no association between use of β-blockers and BC-specific survival in the analyses stratified by PR receptor status, HER2 receptor status, or Ki-67. When stratified by stage, use of any type of β-blockers was associated with BC-specific survival only among patients with localised BC (HR = 1.27, 95% CI: 1.01–1.61) (Fig. 2). Within TNBC patients, use of any type of β-blockers was associated with improved BC-specific survival only in patients with regional cancer (HR = 0.54, 95% CI: 0.34–0.86).

The association between use of any β-blockers and BC-specific survival in patients with TNBC was confirmed in sensitivity analyses where the reference group was non-users of β-blockers who used other antihypertensive medications (HR = 0.66, 95% CI: 0.45–0.95), and when the exposure assessment window was four (HR = 0.65, 95% CI: 0.47–0.90) or 6 months (HR = 0.67, 95% CI: 0.49–0.92). In TNBC patients, multiple imputation and complete-case analyses confirmed the association observed in the main analysis (HR = 0.67, 95% CI: 0.49–0.92; and HR = 0.64, 95% CI: 0.45–0.90, respectively). We found no association between use of other antihypertensive medications and TNBC prognosis.

Kaplan–Meier survival estimates for the use of β-blockers in TNBC patients, stratified by age, are presented in Supplementary Fig. 2.

### Systematic review and meta-analysis

To provide a summary estimate of the association between use of β-blockers and BC prognosis based on the available literature, we conducted a systematic review and meta-analysis of observational studies (including the original cohort described here) that reported molecular subtype-specific estimates of the association. We identified 92 articles, of which 23 articles were fully assessed (Supplementary Fig. 3). Of these, we excluded 14 articles because they did not report molecular subtype-specific estimates [12, 41–53]. Nine articles were included in the systematic review (Table 2) [14–20, 54, 55]. All studies included patients from North America, Europe or Asia. The study by Modi et al. also included patients from South America and Australia/Pacific [18]. The studies by Liu et al. and Modi et al. included patients with HER2 + receptor status but did not contain sufficient information on ER/PR status to distinguish between HER2 + subtype and luminal subtype [18, 55], and so were not included in the pooled estimates. The study by Spera et al. reported estimates from two independent study cohorts and was analysed as two independent studies [17]. In addition, we included the estimates from our original cohort study in the meta-analysis. Hence, the meta-analysis included estimates from nine different cohorts (reported in eight studies). All studies, apart from Liu et al. [55] and Spera et al. [17], were of high quality. The quality of the studies by Liu et al. [55] and Spera et al. [17] were difficult to determine due to limited reporting of important information.

**Table 2:** Descriptive characteristics of studies included in the systematic review.

First author, ref.	Year	Study design	Source of data	Country	Inclusion years	Stage	Age restriction	#Individuals	Molecular subtype	Outcome(s)
Melhem-Bertrandt et al. [14]	2011	Cohort study	Hospital database (MD Anderson Cancer Centre)	USA	1995–2007	Stages I–IV	No	377	TNBC	RFS
Botteri et al. [54]	2013	Cohort study	Hospital database (European Institute of Oncology)	Italy	1997–2008	Stages I–III		800	TNBC	PFS
							Post-menopausal			BCSS
Sørensen et al. [15]	2013	Cohort study	Cancer Registry of Denmark (Nationwide and population-based)	Denmark	1996–2003	Stages I–IV	No	14,200	Luminal (ER+)	RFS
Cardwell et al. [16]	2013	Nested case-control	Clinical Practice Research Datalink, National Cancer Data Repository	UK	1998–2007	Stages I–IV	No	5757	Luminal (ER+)	BCSS
Liu et al. [55]	2016	Cohort study	Hospital database (General Hospital of the People´s Liberation Army)	China	2006–2010	Stage IV	No	94	HER2+ (unknown ER/PR status)	PFS
Spera et al. [17]	2017	Posthoc from RCT	ROSE/TRIO-012 trial	Canada	2008–2011	Stage IV	NR	NR	Luminal (ER+)	PFS
								NR	TNBC	PFS
Spera et al. [17]	2017	Posthoc from RCT	BCRIG-005 trial	Canada	2000–2003	Node positive	NR	35	TNBC	RFS
Santala et al. [20]	2020	Cohort study	Cancer Registry of Finland (Nationwide and population-based)	Finland	1995–2013	Stages I–IV		NR	Luminal (HER−)	BCSS
							No	NR	HER2+	
								NR	TNBC	
Modi et al. [18]	2020	Posthoc from RCT	EMILIA, THERESA, CLEOPATRA, MARIANNE trials	Multi-centre		Stage IV	NR	2777	HER2+ (unknown ER/PR status)	PFS
Lorona et al. [19]	2021	Cohort study	SEER cancer registries (metropolitan area of Albuquerque in New Mexico, and greater metropolitan area of Seattle in Washington)	USA	2004–2015	Stages I–IV		2383	Luminal	RFS
										BCSS
								615	HER2+	BCSS
							20–69 years	1559	TNBC	RFS
										BCSS

*RCT* randomised controlled trial, *UK* United Kingdom, *BC* breast cancer, *TNBC* triple-negative breast cancer, *HER2* human epidermal growth factor receptor 2, *ER* oestrogen receptor, *PR* progesterone receptor, *RFS* recurrence-free survival, *PFS* progression-free survival, *BCSS* breast cancer-specific survival, *NR* not reported.

**Fig. 3:**
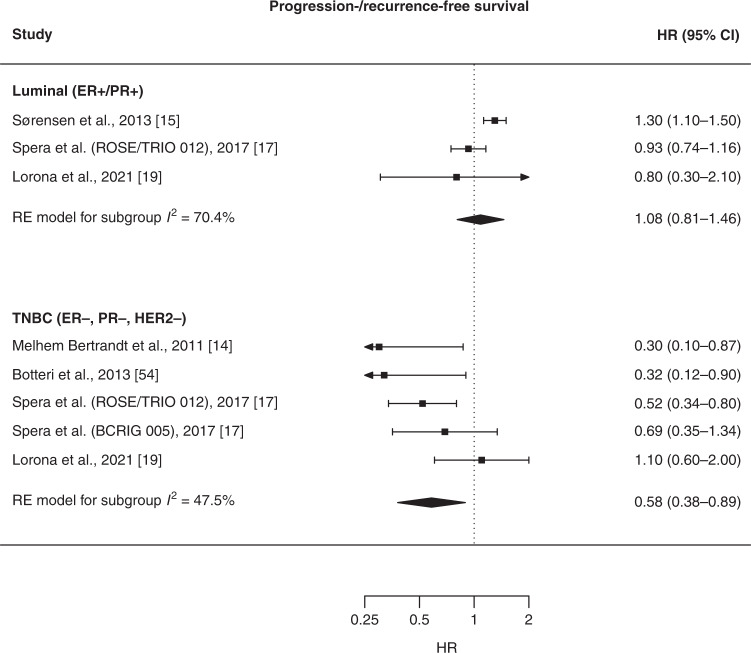
Forest plot of estimates for the association between use of β-blockers and progression-free or recurrence-free survival, stratified by molecular subtype. Comparison group is users of other antihypertensive medications in the study by Lorona et al. [19] and all non-users of β-blockers in the other studies. HR hazard ratio, CI confidence interval, ER oestrogen receptor, PR progesterone receptor, HER2 human epidermal growth factor receptor 2, RE random effect, TNBC triple-negative breast cancer.

**Fig. 4:**
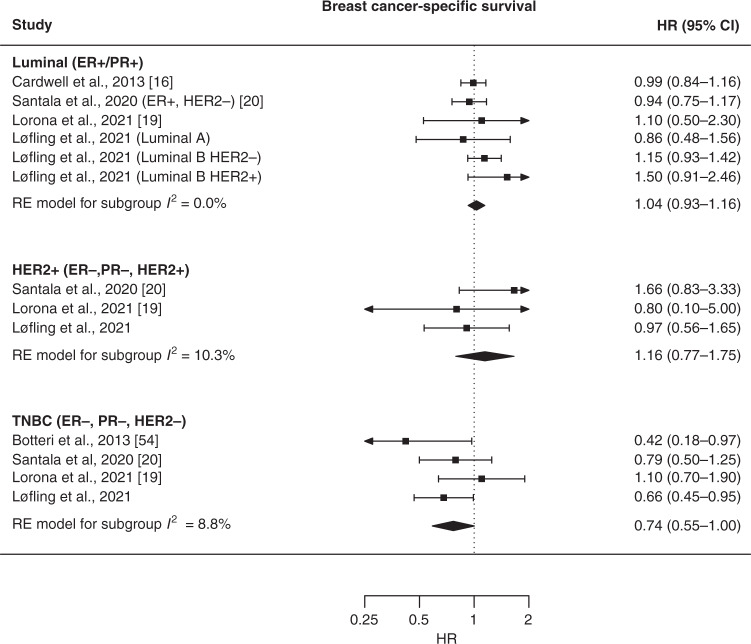
Forest plot of estimates for the association between use of β-blockers breast cancer-specific survival, stratified by molecular subtype. Comparison group is users of other antihypertensive medications in the study by Lorona et al. [19] and in our original cohort study, and all non-users of β-blockers in the other studies. HR hazard ratio, CI confidence interval, ER oestrogen receptor, PR progesterone receptor, HER2 human epidermal growth factor receptor 2, RE random effect, TNBC triple-negative breast cancer.

In the meta-analysis for PFS/RFS, HRs for use of β-blockers compared to no use were 1.08 (95% CI: 0.81–1.46) for luminal BC (combining luminal A and luminal B) and 0.58 (95% CI: 0.38–0.89) for TNBC (Fig. 3). None of the included studies reported estimates for PFS/RFS in patients with HER2+ BC. In the meta-analysis for BC-specific survival, HRs for use of β-blockers compared to no use were 1.04 (95% CI: 0.93–1.16) for luminal BC (combining luminal A and luminal B), 1.16 (95% CI: 0.77–1.75) for HER2+ BC, and 0.74 (95% CI: 0.55–1.00) for TNBC (Fig. 4). We tested publication bias for TNBC estimates and found no evidence of publication bias (PFS/RFS: *p* value = 0.52, BC-specific survival: *p* value = 0.80) (Supplementary Figs. 4, 5).

In a sensitivity analysis we changed the estimates from the study by Lorona et al. [19] and from the original cohort study reported here to estimates that compared users of β-blockers to all non-users of β-blockers, while the estimates from the other studies remained the same (i.e. having all non-users of β-blockers as comparison group). In this analysis, the HRs for use of β-blockers compared to no use for PFS/RFS were 1.13 (95% CI: 0.86–1.49) for luminal BC (combining luminal A and luminal B) and 0.60 (95% CI: 0.36–1.00) for TNBC, while for BC-specific survival, HRs were 1.03 (95% CI: 0.93–1.13) for luminal BC (combing luminal A and luminal B), 1.19 (95% CI: 0.84–1.70) for HER2+ BC, and 0.78 (95% CI: 0.52–1.16) for TNBC.

## Discussion

The findings from this cohort study and meta-analysis suggest that β-blockers may exert differential effects on survival depending on women’s BC molecular subtypes. In our original cohort study, we found evidence that use of β-blockers are associated with a 34% reduction in BC mortality in TNBC patients, while we found no evidence of an association with reduced BC mortality in any of the other molecular subtypes, or in the overall population. These findings were supported by the meta-analysis.

These findings may reconcile inconsistent results that have been found across a growing number of studies that reported on use of β-blockers and prognosis in BC cohorts. While a number of studies have found that β-blockers are associated with improved BC prognosis in a general BC population [12, 44, 50, 56], many studies found no association [16, 41, 43, 46–49, 51]. By not stratifying by molecular subtype, these latter studies may have missed an opportunity to identify subsets of patients who experience improved outcomes after β-blocker use.

Consistent with our finding, a number of studies that focused on TNBC have reported an association between β-blocker use and improved BC prognosis. Melhem-Bertrandt et al. studied 377 TNBC patients in the USA and reported a longer RFS associated with use of β-blockers (HR = 0.30, 95% CI: 0.10–0.87) [14]. Botteri et al. studied 800 TNBC patients in Italy and estimated longer BC-specific survival associated with use of β-blockers (HR = 0.42, 95% CI: 0.18–0.97) [54]. Spera et al. performed a retrospective analysis of data from two Canadian clinical trials (ROSE/TRIO-12 and BCRIG-005) and reported, in TNBC patients, an improved PFS associated with use of β-blockers (HR = 0.52, 95% CI: 0.34–0.80) in the ROSE/TRIO-12 trial, and an indication of that use of β-blockers may be associated with RFS in the BCRIG-005 trial (HR = 0.69, 95% CI: 0.35–1.34) [17]. The results of an association between use of β-blockers and improved BC-specific survival for patients with TNBC in our study are also in line with a previous meta-analysis that focused on patients with early BC only [57]. However, two studies did not find an association in TNBC patients [19, 20]. Santala et al. found an indication of improved BC-specific survival (HR = 0.79, 95% CI: 0.50–1.25) [20], while Lorona et al. reported HRs of 1.10 (95% CI: 0.60–2.00) for RFS and 1.10 (95% CI: 0.70–1.90) for BC-specific survival [19].

The findings of no association between use of β-blockers and BC-specific survival in patients with BC of luminal or HER2 + molecular subtypes in our cohort study are consistent with previous observational studies [16, 17, 19, 20] and was confirmed in the meta-analysis. However, a large Danish registry-based cohort study by Sørensen et al. that included patients with luminal BC (ER+), reported that β-blocker use was associated with shorter RFS (HR = 1.30, 95% CI: 1.10–1.50) [15]. Modi et al. performed a retrospective analysis of four clinical trials that enroled HER2+ BC patients (unknown ER/PR status) and found an indication of a shorter PFS associated with the use of β-blockers (HR = 1.10, 95% CI: 0.92–1.30) [18], similar to our findings for BC-specific survival in patients with HER2+ receptor status. In contrast, preclinical studies suggest that β-blockers may re-sensitise cancer cells to anti-HER2 therapy, which may be predicted to improve BC prognosis [55]. The same paper reported an indication of improved outcome associated with use of β-blockers in a clinical cohort of patients with HER2+ BC (unknown ER/PR status) treated with trastuzumab and chemotherapy. However, a clinical study indicated that a high expression of β_2_-adrenergic receptors may be associated with improved prognosis in early BC patients with HER2+ receptor status [58], indicating a potential decrease in BC prognosis when blocking the β_2_-adrenergic receptor in HER2+ patients. Future mechanistic studies are required to understand the role of β-adrenergic signalling—and β-blockade—in HER2+ patients.

The time period surrounding cancer diagnosis and treatment is stressful for the patient [56, 59]. Stress elevates the activity of the sympathetic nervous system and can increase cancer progression via β-adrenergic signalling [9]. Mechanistic studies revealed that activation of β-adrenergic signalling in tumour cells enhances tumour cell invasion by remodelling their cytoskeleton to create invasive structures called invadopodia [60, 61]. Preclinical studies have also shown that activation of β-adrenergic signalling shapes the tumour microenvironment by promoting the formation of blood and lymph vessels [7–9] and inducing immunosuppression [62], which supports cancer progression. Blocking β-adrenergic signalling using β-blockers effectively inhibited the effects of stress and improved cancer outcomes in mouse models of BC, mainly of TNBC [7, 9, 62]. The findings from our present study are consistent with these mechanistic studies, and suggest that use of β-blockers during diagnosis and treatment could improve TNBC prognosis.

The biological reason behind the association between β-blocker use and improved prognosis for TNBC patients but not for other BC patients remains unclear. TNBC is more immunogenic than other subtypes of BC [63]. As a consequence, TNBC may be more sensitive than other subtypes to restoration of anti-cancer immunity by treatment with β-blockers [5, 62, 63]. In addition, patients with TNBC receive chemotherapy more often than patients with other types of BC. Stress has been suggested to modulate chemotherapy efficacy through β-adrenergic signalling in preclinical studies [30, 31, 64]. Therefore, to the extent that β-blockers exert a therapeutic effect through a synergistic interaction with chemotherapy, this may be less relevant to patients with other types of BC.

The lack of an association between use of β-blockers and BC-specific survival among patients with localised TNBC may be explained by the fact that the prognosis for patients with localised cancer is generally good [26], and the use of β-blockers might have no measurable impact on the prognosis for these patients. The positive association found in the overall population of BC patients with localised disease is driven by patients with luminal or HER2 + BC. In TNBC patients, we found evidence of an association between use of β-blockers and BC-specific survival in patients with regional cancer, but not with advanced cancer. The absence of an association between use of β-blockers and BC-specific survival in patients with advanced cancer may be because β-blockers were used too late in cancer progression to have an observable effect on prognosis, or because of limited statistical power in our cohort.

The classification of β-blockers as selective or non-selective is based on their affinity for different subtypes of the β-adrenergic receptors, with selective β-blockers having a higher affinity for the β_1_-adrenergic receptor subtype than the other subtypes [65]. While preclinical studies have identified an important role for the β_2_-adrenergic receptor in cancer progression [9], the role of the β_1_-adrenergic receptor is less clear. Our findings that both selective and non-selective β-blockers are associated with improved BC-specific survival in TNBC suggest that further mechanistic studies are needed to understand the role of the various receptor subtypes.

### Strengths and limitations

The main strength of our cohort study is the population-based design, with data retrieved from nationwide registries of high quality, which reduced the risk of misclassification bias. The Cancer Registry of Norway includes data on all women with BC in Norway, resulting in no risk of selection bias. The use of a prescription database avoided self-reported use of drugs, which may be less accurate and associated with a higher risk of introducing misclassification bias. Another important strength was the large sample size, which allowed stratification by molecular subtype and other prognostic factors. Notably, we obtained robust results in the different sensitivity analyses. However, there are several limitations. The Norwegian Prescription Database records information on filled prescriptions from 2004 onwards, therefore we could not look at filled prescriptions before that. Also, the database contains no information on actual use of the medications from the filled prescriptions. Non-compliance may have biased HRs towards no association. In addition, the database contains no information on the indication for the prescription, potentially introducing confounding by indication. However, in Norway β-blockers are prescribed mainly for hypertension, and the sensitivity analysis where the comparison group included only users of other antihypertensive medications gave similar estimates, indicating that confounding by indication is not of substantial concern. We did not have access to information on comorbid conditions. However, this was partially addressed by using filled prescriptions as proxy for comorbidities. Multiple testing might be a concern, but the fact that the results from the original cohort study were confirmed in the meta-analysis may somewhat alleviate this concern. A further limitation of the original cohort study is that we cannot generalise our results to women aged <50 years, a subgroup of special interest in TNBC. However, the original cohort included only 13 TNBC patients aged <50 years who used β-blockers making a subgroup analysis in young women difficult. The main limitation of the meta-analysis is that only a small number of studies reported molecular subtype-specific estimates.

## Conclusion

In a large population-based cohort study we found evidence of an association between use of β-blockers at diagnosis and decreased BC mortality in patients with TNBC. A meta-analysis of available literature confirmed these findings.

### Reporting summary

Further information on research design is available in the Nature Research Reporting Summary linked to this article.

## Supplementary information

Supplementary material

Reporting summary

PRISMA guideline

STROBE guideline

## Data Availability

Due to Norwegian law, we are not allowed to make the data publicly available. However, the data can be requested from the registry holders.

## Code availability

The code used for data management and analysis can be obtained from the corresponding author upon reasonable request.

## References

[1] Sung H, Ferlay J, Siegel RL, Laversanne M, Soerjomataram I, Jemal A (2021). Global Cancer Statistics 2020: GLOBOCAN Estimates of Incidence and Mortality Worldwide for 36 Cancers in 185 Countries. CA Cancer J Clin.

[2] Sebuødegård S, Botteri E, Hofvind S (2019). Breast cancer mortality after implementation of organized population-based breast cancer screening in Norway. J Natl Cancer Inst.

[3] Cancer Registry of Norway. Cancer in Norway 2020—Cancer incidence, mortality, survival and prevalence in Norway. Oslo, Norway: Caner Registry of Norway; 2021.

[4] Johansson ALV, Trewin CB, Hjerkind KV, Ellingjord-Dale M, Johannesen TB, Ursin G (2019). Breast cancer-specific survival by clinical subtype after 7 years follow-up of young and elderly women in a nationwide cohort. Int J Cancer.

[5] Nissen MD, Sloan EK, Mattarollo SR (2018). β-Adrenergic signaling impairs antitumor CD8+ T-cell responses to B-cell lymphoma immunotherapy. Cancer Immunol Res.

[6] Mohammadpour H, MacDonald CR, McCarthy PL, Abrams SI, Repasky EA. β2-adrenergic receptor signaling regulates metabolic pathways critical to myeloid-derived suppressor cell function within the TME. Cell Rep. 2021. 10.1016/j.celrep.2021.109883. 10.1016/j.celrep.2021.109883 PMC8601406 34706232

[7] Le CP, Nowell CJ, Kim-Fuchs C, Botteri E, Hiller JG, Ismail H, et al. Chronic stress in mice remodels lymph vasculature to promote tumour cell dissemination. Nature Commun. 2016. 10.1038/ncomms10634. 10.1038/ncomms10634 PMC4773495 26925549

[8] Thaker PH, Han LY, Kamat AA, Arevalo JM, Takahashi R, Lu C (2006). Chronic stress promotes tumor growth and angiogenesis in a mouse model of ovarian carcinoma. Nat Med.

[9] Sloan EK, Priceman SJ, Cox BF, Yu S, Pimentel MA, Tangkanangnukul V (2010). The sympathetic nervous system induces a metastatic switch in primary breast cancer. Cancer Res.

[10] Chang A, Le CP, Walker AK, Creed SJ, Pon CK, Albold S (2016). 2-Adrenoceptors on tumor cells play a critical role in stress-enhanced metastasis in a mouse model of breast cancer. Brain Behav Immun.

[11] Cole SW, Sood AK (2012). Molecular pathways: beta-adrenergic signaling in cancer. Clin Cancer Res.

[12] Gillis RD, Botteri E, Chang A, Ziegler AI, Chung NC, Pon CK (2021). Carvedilol blocks neural regulation of breast cancer progression in vivo and is associated with reduced breast cancer mortality in patients. Eur J Cancer.

[13] Zhong S, Yu D, Zhang X, Chen X, Yang S, Tang J (2016). β-Blocker use and mortality in cancer patients: systematic review and meta-analysis of observational studies. Eur J Cancer Prev.

[14] Melhem-Bertrandt A, Chavez-Macgregor M, Lei X, Brown EN, Lee RT, Meric-Bernstam F (2011). Beta-blocker use is associated with improved relapse-free survival in patients with triple-negative breast cancer. J Clin Oncol.

[15] Sørensen GV, Ganz PA, Cole SW, Pedersen LA, Sørensen HT, Cronin-Fenton DP (2013). Use of β-blockers, angiotensin-converting enzyme inhibitors, angiotensin II receptor blockers, and risk of breast cancer recurrence: a Danish nationwide prospective cohort study. J Clin Oncol.

[16] Cardwell CR, Coleman HG, Murray LJ, Entschladen F, Powe DG (2013). Beta-blocker usage and breast cancer survival: a nested case-control study within a UK Clinical Practice Research Datalink cohort. Int J Epidemiol.

[17] Spera G, Fresco R, Fung H, Dyck JRB, Pituskin E, Paterson I (2017). Beta blockers and improved progression-free survival in patients with advanced HER2 negative breast cancer: a retrospective analysis of the ROSE/TRIO-012 study. Ann Oncol.

[18] Modi ND, Tan JQE, Rowland A, Koczwara B, Kichenadasse G, McKinnon RA, et al. The influence of pre-existing beta-blockers use on survival outcomes in HER2 positive advanced breast cancer: pooled analysis of clinical trial data. Front Oncol. 2020. 10.3389/fonc.2020.01130. 10.3389/fonc.2020.01130 PMC7373122 32760671

[19] Lorona NC, Cook LS, Tang M-TC, Hill DA, Wiggins CL, Li CI (2021). Antihypertensive medications and risks of recurrence and mortality in luminal, triple-negative, and HER2-overexpressing breast cancer. Cancer Causes Control.

[20] Santala EEE, Murto MO, Artama M, Pukkala E, Visvanathan K, Murtola TJ (2020). Angiotensin receptor blockers associated with improved breast cancer survival—a nationwide cohort study from Finland. Cancer Epidemiol Biomark Prev.

[21] Norwegian Tax Agency. Personal Identification Number. 2021. https://www.skatteetaten.no/en/person/foreign/norwegian-identification-number/what-is-an-identification-number/. Accessed 2021-08-02.

[22] Larsen IK, Smastuen M, Johannesen TB, Langmark F, Parkin DM, Bray F (2009). Data quality at the Cancer Registry of Norway: an overview of comparability, completeness, validity and timeliness. Eur J Cancer.

[23] Elston CW, Ellis IO (1991). Pathological prognostic factors in breast cancer. I. The value of histological grade in breast cancer: experience from a large study with long-term follow-up. Histopathol.

[24] Sisti JS, Collins LC, Beck AH, Tamimi RM, Rosner BA, Eliassen AH (2016). Reproductive risk factors in relation to molecular subtypes of breast cancer: Results from the nurses’ health studies. Int J Cancer.

[25] National Cancer Institute. Surveillance, Epidemiology, and End Results (SEER) Program. 2021. https://seer.cancer.gov/ Accessed 2021-12-01.

[26] American Joint Committee on Cancer. AJCC cancer staging manual. 7th edn. New York, USA: Springer Verlag New York Inc; 2010.

[27] Furu, K Establishment of the nationwide Norwegian Prescription Database (NorPD)–new opportunities for research in pharmacoepidemiology in Norway. Norsk Epidemiol. 2009;18. 10.5324/nje.v18i2.23.

[28] Norwegian Institute of Public Health. Norwegian Cause of Death Registry. 2020. https://www.fhi.no/hn/helseregistre-og-registre/dodsarsaksregisteret/dodsarsaksregisteret2/. Accessed 2021-06-04.

[29] Statistics Norway. https://www.ssb.no/en. 2021. Accessed 2021-12-01.

[30] Pasquier E, Ciccolini J, Carre M, Giacometti S, Fanciullino R, Pouchy C (2011). Propranolol potentiates the anti-angiogenic effects and anti-tumor efficacy of chemotherapy agents: implication in breast cancer treatment. Oncotarget.

[31] Reeder A, Attar M, Nazario L, Bathula C, Zhang A, Hochbaum D (2015). Stress hormones reduce the efficacy of paclitaxel in triple negative breast cancer through induction of DNA damage. Br J Cancer.

[32] Korzeniowski S, Dyba T (1994). Reproductive history and prognosis in patients with operable breast cancer. Cancer.

[33] Zhai Z, Zhang F, Zheng Y, Zhou L, Tian T, Lin S (2019). Effects of marital status on breast cancer survival by age, race, and hormone receptor status: a population-based Study. Cancer Med.

[34] Lundqvist A, Andersson E, Ahlberg I, Nilbert M, Gerdtham U (2016). Socioeconomic inequalities in breast cancer incidence and mortality in Europe-a systematic review and meta-analysis. Eur J Public Health.

[35] Cronin-Fenton DP, Nørgaard M, Jacobsen J, Garne JP, Ewertz M, Lash TL (2007). Comorbidity and survival of Danish breast cancer patients from 1995 to 2005. Br J Cancer.

[36] van Buuren S, Groothuis-Oudshoorn K (2011). Mice: multivariate imputation by chained equations in R. J Stat Softw.

[37] van Houwelingen HC, Arends LR, Stijnen T (2002). Advanced methods in meta-analysis: multivariate approach and meta-regression. Stat Med.

[38] Higgins JPT, Thompson SG (2002). Quantifying heterogeneity in a meta-analysis. Stat Med.

[39] Egger M, Smith GD, Schneider M, Minder C (1997). Bias in meta-analysis detected by a simple, graphical test. BMJ.

[40] Wells GA, Shea B, O’Connell D, Peterson J, Welch V, Losos M, et al. The Newcastle-Ottawa Scale (NOS) for Assessing the Quality of Nonrandomised Studies in Meta-Analyses. 2008. http://www.ohri.ca/programs/clinical_epidemiology/oxford.asp. Accessed 2022-01-07.

[41] Barron TI, Connolly RM, Sharp L, Bennett K, Visvanathan K (2011). Beta blockers and breast cancer mortality: a population- based study. J Clin Oncol.

[42] Boudreau DM, Yu O, Chubak J, Wirtz HS, Bowles EJA, Fujii M (2014). Comparative safety of cardiovascular medication use and breast cancer outcomes among women with early stage breast cancer. Breast Cancer Res Treat.

[43] Cardwell CR, Pottegård A, Vaes E, Garmo H, Murray LJ, Brown C (2016). Propranolol and survival from breast cancer: a pooled analysis of European breast cancer cohorts. Breast Cancer Res.

[44] Chae YK, Brown EN, Lei X, Melhem-Bertrandt A, Giordano SH, Litton JK (2013). Use of ACE inhibitors and angiotensin receptor blockers and primary breast cancer outcomes. J Cancer.

[45] Chen L, Chubak J, Boudreau DM, Barlow WE, Weiss NS, Li CI (2017). Use of antihypertensive medications and risk of adverse breast cancer outcomes in a SEER-medicare population. Cancer Epidemiol Biomark Prev.

[46] Ganz PA, Habel LA, Weltzien EK, Caan BJ, Cole SW (2011). Examining the influence of beta blockers and ACE inhibitors on the risk for breast cancer recurrence: results from the LACE cohort. Breast Cancer Res Treat.

[47] Holmes S, Griffith EJ, Musto G, Minuk GY (2013). Antihypertensive medications and survival in patients with cancer: A population-based retrospective cohort study. Cancer Epidemiol.

[48] Kreklau A, Nel I, Kasimir-Bauer S, Kimmig R, Frackenpohl AC, Aktas B (2021). An observational study on breast cancer survival and lifestyle related risk factors. Vivo.

[49] Musselman RP, Bennett S, Li W, Mamdani M, Gomes T, van Walraven C (2018). Association between perioperative beta blocker use and cancer survival following surgical resection. Eur J Surg Oncol.

[50] Powe DG, Voss MJ, Zänker KS, Habashy HO, Green AR, Ellis IO (2010). Beta-blocker drug therapy reduces secondary cancer formation in breast cancer and improves cancer specific survival. Oncotarget.

[51] Sakellakis M, Kostaki A, Starakis I, Koutras A (2014). β-Blocker use and risk of recurrence in patients with early. Breast Cancer Chemother.

[52] Shah SM, Carey IM, Owen CG, Harris T, DeWilde S, Cook DG (2011). Does beta-adrenoceptor blocker therapy improve cancer survival? Findings from a population-based retrospective cohort study. Br J Clin Pharm.

[53] Wong MCS, Tam WWS, Lao X, Wang HHX, Kwan MWM, Cheung CSK (2015). The incidence of cancer deaths among hypertensive patients in a large Chinese population: a cohort study. Int J Cardiol.

[54] Botteri E, Munzone E, Rotmensz N, Cipolla C, De Giorgi V, Santillo B (2013). Therapeutic effect of β-blockers in triple-negative breast cancer postmenopausal women. Breast Cancer Res Treat.

[55] Liu D, Yang Z, Wang T, Yang Z, Chen H, Hu Y (2016). β2-AR signaling controls trastuzumab resistance-dependent pathway. Oncogene.

[56] Hiller JG, Cole SW, Crone EM, Byrne DJ, Shackleford DM, Pang JB (2020). Preoperative β-Blockade with Propranolol Reduces Biomarkers of Metastasis in Breast Cancer: A Phase II Randomized Trial. Clin Cancer Res.

[57] Caparica R, Bruzzone M, Agostinetto E, De Angelis C, Fêde Â, Ceppi M et al. Beta-blockers in early-stage breast cancer: a systematic review and meta-analysis. ESMO Open. 2021;6. 10.1016/j.esmoop.2021.100066. 10.1016/j.esmoop.2021.100066 PMC7921512 33639601

[58] Caparica R, Richard F, Brandão M, Awada A, Sotiriou C, de Azambuja E (2020). Prognostic and predictive impact of beta-2 adrenergic receptor expression in HER2-positive breast cancer. Clin Breast Cancer.

[59] Hiller JG, Perry NJ, Poulogiannis G, Riedel B, Sloan EK (2018). Perioperative events influence cancer recurrence risk after surgery. Nat Rev Clin Oncol.

[60] Creed SJ, Le CP, Hassan M, Pon CK, Albold S, Chan KT (2015). 2-adrenoceptor signaling regulates invadopodia formation to enhance tumor cell invasion. Breast Cancer Res.

[61] Kim TH, Gill NK, Nyberg KD, Nguyen AV, Hohlbauch SV, Geisse NA (2016). Cancer cells become less deformable and more invasive with activation of β-adrenergic signaling. J Cell Sci.

[62] Bucsek MJ, Qiao G, MacDonald CR, Giridharan T, Evans L, Niedzwecki B (2017). β-adrenergic signaling in mice housed at standard temperatures suppresses an effector phenotype in CD8(+) T cells and undermines checkpoint inhibitor therapy. Cancer Res.

[63] Kwa MJ, Adams S (2018). Checkpoint inhibitors in triple-negative breast cancer (TNBC): where to go from here. Cancer.

[64] Pasquier E, Street J, Pouchy C, Carre M, Gifford AJ, Murray J (2013). β-blockers increase response to chemotherapy via direct antitumour and anti-angiogenic mechanisms in neuroblastoma. Br J Cancer.

[65] Gorre F, Vandekerckhove H (2010). Beta-blockers: focus on mechanism of action. Which beta- blocker, when and why?. Acta Cardiol.

