# Supplementary material for: β-blockers and breast cancer survival by molecular subtypes: a population-based cohort study and meta-analysis
Source: Br J Cancer. 2022 Jun 20;127(6):1086–96. doi: 10.1038/s41416-022-01891-7 (PMC9470740; doi:10.1038/s41416-022-01891-7)
Supplement: Supplementary file 1 — Supplementary material [file 41416_2022_1891_MOESM1_ESM.docx]

**Supplementary material**

| Supplementary Table 1. Co-medications and corresponding Anatomical Therapeutic Anatomy (ATC) codes. | |
| --- | --- |
| Medication | ATC-code |
| Angiotensin converting enzyme inhibitors (ACEI) | C09A, C09B |
| Angiotensin receptor blockers (ARB) | C09C, C09D |
| Calcium channel blockers (CCB) | C08, C09BB, C09DB |
| Diuretics | C03, C07B, C09BA, C09DA |
| Low dose aspirin | B01AC06, B01AC56 |
| Cyclooxygenase2 (COX2) inhibitors | M01AH |
| Statins | C10AA, C10BA02, C10BA05, C10BA06 |
| Antidiabetics | A10 |

| Supplementary Table 2. Search strings used in the systematic review. | |
| --- | --- |
| PubMed | |
| Breast cancer | *((((breast neoplasms[MeSH Terms]) OR (breast cancer[Title/Abstract])) OR (breast tumor[Title/Abstract])) OR (breast carcinoma[Title/Abstract])) OR (breast neoplasm[Title/Abstract])* |
| Β-blocker | *((((((((((Adrenergic beta-Antagonists[MeSH Terms]) OR (adrenergic beta antagonists[Title/Abstract])) OR (beta blockers[Title/Abstract])) OR (propranolol[Title/Abstract])) OR (timolol[Title/Abstract])) OR (metoprolol[Title/Abstract])) OR (atenolol[Title/Abstract])) OR (bisoprolol[Title/Abstract])) OR (carvedilol[Title/Abstract])) OR (labetalol[Title/Abstract])) OR (pindolol[Title/Abstract])* |
| Observational study | *(((((Cohort Studies[MeSH Terms])) OR (Case-Control Studies[MeSH Terms])) OR (cohort study[Title/Abstract])) OR (case-control study[Title/Abstract])) OR (observational study[Title/Abstract])* |
| Combined search | #1 AND #2 AND #3  56 hits (2021-09-14) |
| Web of Science | |
| Breast cancer | *(((((((TI=(breast cancer)) OR TI=(breast tumor)) OR TI=(breast carcinoma)) OR TI=(breast neoplasm)) OR AB=(breast cancer)) OR AB=(breast tumor)) OR AB=(breast carcinoma)) OR AB=(breast neoplasm)* |
| Β-blocker | *(((((((((((((((((((TI=(adrenergic beta antagonists)) OR TI=(beta blockers)) OR TI=(propranolol)) OR TI=(timolol)) OR TI=(metoprolol)) OR TI=(atenolol)) OR TI=(bisoprolo)) OR TI=(carvedilol)) OR TI=(labetalol)) OR TI=(pindolol)) OR AB=(adrenergic beta antagonists)) OR AB=(beta blockers)) OR AB=(propranolol)) OR AB=(timolol)) OR AB=(metoprolol)) OR AB=(atenolol)) OR AB=(bisoprolol)) OR AB=(carvedilol)) OR AB=(labetalol)) OR AB=(pindolol)* |
| Observational study | *(((((TI=(cohort study)) OR TI=(case-control study)) OR TI=(observational study)) OR AB=(cohort study)) OR AB=(case-control study)) OR AB=(observational study)* |
| Combined search | #1 AND #2 AND #3  54 hits (2021-09-14) |


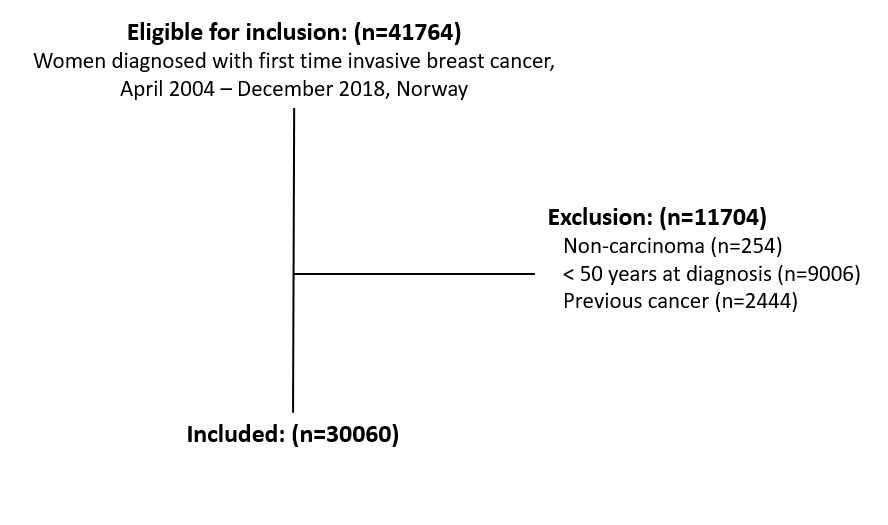


Supplementary Figure 1. Flow chart of inclusion of breast cancer patients, April 2004 – December 2018, Norway


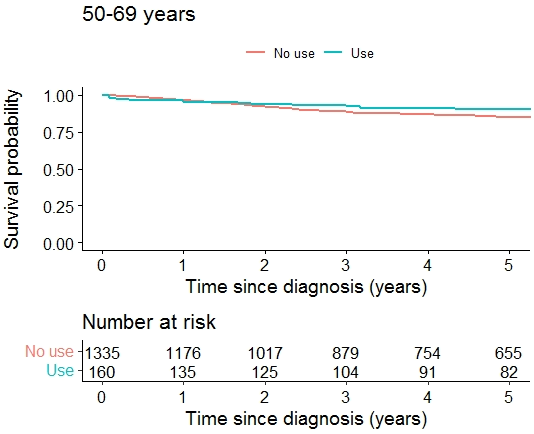

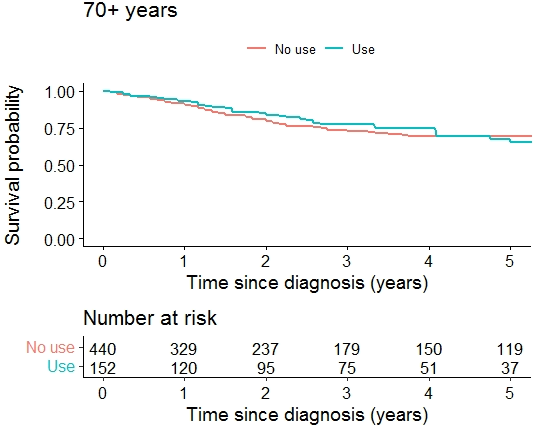


Supplementary Figure 2. Kaplan-Meier survival curves for triple negative breast cancer-specific survival by use of β-blockers, stratified by age.


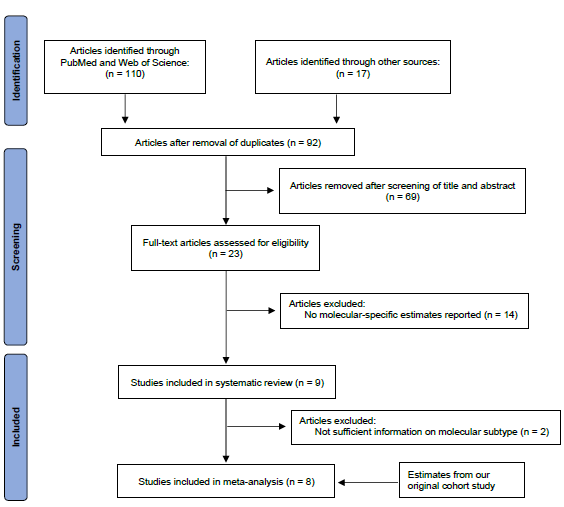


Supplementary Figure 3. PRIMSA flow chart for inclusion of studies in the systematic review and meta-analysis.


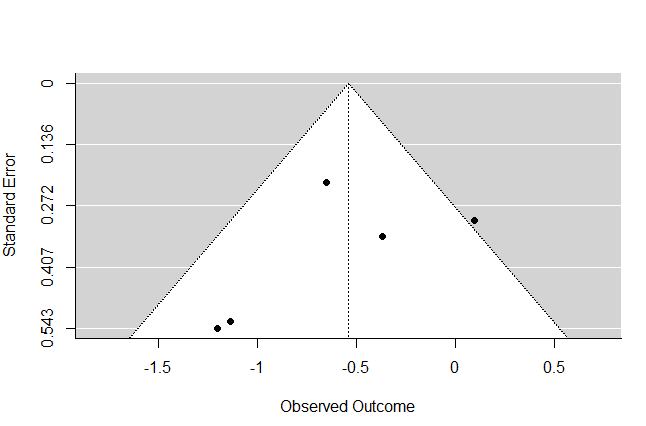


Supplementary Figure 4. Funnel plot of studies assessing the association between use of β-blockers and progression-free or recurrence-free survival in patients with triple negative breast cancer.


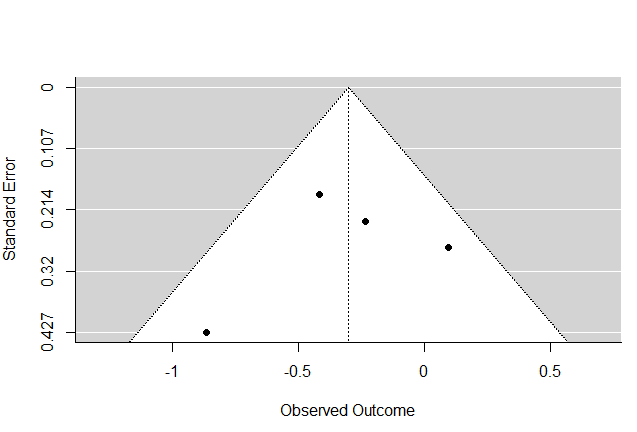


Supplementary Figure 5. Funnel plot of studies assessing the association between use of β-blockers and breast cancer-specific survival in patients with triple negative breast cancer.
